# A randomised trial evaluating the effect of intraoperative iron administration

**DOI:** 10.1038/s41598-020-72827-5

**Published:** 2020-09-28

**Authors:** Bora Lee, Eun Jung Kim, Jaewoo Song, Young-Soo Jung, Bon-Nyeo Koo

**Affiliations:** 1 Department of Anesthesiology and Pain Medicine, Severance Hospital and Anesthesia and Pain Research Institute, Yonsei University College of Medicine, 50-1 Yonsei-ro, Seodaemun-Gu, Seoul, 03722 Republic of Korea; 2 Department of Laboratory Medicine, Yonsei University College of Medicine, Yonsei-ro 50-1, Seodaemun-gu, Seoul, 03722 Republic of Korea; 3 Department of Oral & Maxillofacial Surgery, College of Dentistry, Yonsei University, 50-1 Yonsei-ro, Seodaemun-gu, Seoul, 03722 Republic of Korea

**Keywords:** Risk factors, Medical research

## Abstract

Perioperative anaemia increases postoperative morbidity and mortality, and iron deficiency is anaemia’s most common cause in surgical patients. Preoperative intravenous iron increases postoperative haemoglobin; however, data regarding intraoperative intravenous iron’s effectiveness are inadequate. This study examined intraoperative intravenous iron’s effects on postoperative haemoglobin levels in adults. Fifty-seven healthy subjects (aged 19–40 years) scheduled for bimaxillary orthognathic surgery were assigned randomly to the iron (n = 28) or control (n = 29) groups. The iron group received intravenous ferric derisomaltose (1,000 mg) after anaesthetic induction. The control group received an identical volume of intravenous normal saline. The primary outcome was postoperative haemoglobin level. Secondary outcomes included other postoperative haematologic and iron parameters. Laboratory data were obtained preoperatively and at 1 day, 2 weeks, and 4 weeks postoperatively. Haemoglobin was higher in the iron group 2 weeks postoperatively (12.9 g/dL vs. 12.2 g/dL), but the between-group difference was not significant after adjustment for multiple testing. However, the reticulocyte production index was significantly higher in the iron group 2 weeks postoperatively. Intraoperative intravenous iron maintains postoperative haemoglobin values in patients undergoing bimaxillary orthognathic surgery by increasing haematopoietic function and iron bioavailability and therefore appears to be a useful strategy for blood management.

## Introduction

Perioperative anaemia increases postoperative morbidity and mortality^1^. Up to 75% of patients present with anaemia before surgery^2^. Iron deficiency, secondary to intraoperative blood loss and increased iron requirements, is the leading cause of anaemia in surgical patients^3, 4^. Identification of preoperative anaemia and iron deficiency has been recommended for all those with anticipated moderate to high (> 500 mL) surgical blood loss^5, 6^.

Bimaxillary orthognathic surgery is performed for correction of facial abnormalities, such as prognathism, retrognathism, and facial asymmetry^7^. Because of high vascularity and poor visualisation of the surgical site, this surgery is accompanied by a substantial likelihood of excessive blood loss and the potential need for blood transfusion^8, 9^. Preoperative autologous blood donation and hypotensive anaesthesia are often used to reduce the need for allogenic transfusion with bimaxillary orthognathic surgery^10^, but preoperative autologous blood donation has several drawbacks. The donation process can be uncomfortable for the patient, it can lead to anaemia, and its cost is relatively high.

Preoperative intravenous (IV) iron has been demonstrated to reduce transfusion requirements and increase postoperative haemoglobin (Hb) levels^11–16^. Furthermore, there are a number of trials reporting on the improved erythropoietic function (increased reticulocyte count) using preoperative IV iron^11, 17, 18^. However, administration of preoperative IV iron is not always feasible, especially in emergency surgery. Infusing iron intraoperatively is more convenient, but data regarding the effectiveness of this approach are inadequate^19^. We thereby conducted a randomised controlled trial to evaluate the effects of intraoperative IV iron on postoperative Hb levels and transfusion requirements in patients undergoing bimaxillary orthognathic surgery.

## Methods

### Patients

The Severance Hospital Institutional Review Board (Yonsei University Health System, Seoul, Republic of Korea, Chairperson Professor Seung Min Kim) approved the study protocol on 17 February 2017 (No. 4-2016-1146), which is registered at ClinicalTrials.gov (No. NCT03094182, 29/03/17). This study was performed in accordance with the Declaration of Helsinki. All participants provided written informed consent before randomisation. We enrolled 57 adults (aged 19–40 years) who underwent elective bimaxillary orthognathic surgery between September 2017 and March 2019 by one surgeon (Y-S.J.). The American Society of Anesthesiologists (ASA) physical status class of all subjects was I. The exclusion criteria were pregnancy, haematologic disease, kidney-related anaemia, hepatitis, severe atopy, or drug allergies. None received iron supplements. The preoperative autologous blood donation was performed 2 weeks prior to surgery.

### Randomisation and interventions

Participants were assigned at random to one of two groups—iron group (n = 28) or control group (n = 29)—using a computer-generated randomisation table (available at https://www.random.org). An anaesthesiologist not involved in data collection conducted the randomisation and group assignments. The iron group received 1000 mg ferric derisomaltose (Monofer, 200 mg/2 mL, Solupharm, Melsungen, Germany), which was added to normal saline to generate a total volume of 100 mL. This solution was infused over 30 min after anaesthesia induction. The same volume of normal saline was administered in an identical manner to the control group. An anaesthesiologist not involved in data collection prepared the two solutions. The investigator, surgeons, and study participants were blinded to the group assignment.

### Anaesthesia

Anaesthesia was induced with 2 mg kg^−1^ propofol (Fresofol MCT 1%, Fresenius Kabi Korea Ltd, Seoul, Korea) and 0.5–1 µg kg^−1^ remifentanil (Ultiva, GlaxoSmithKline Korea, Seoul, Korea). Tracheal intubation was facilitated with 1.0 mg kg^−1^ rocuronium (Esmeron, MSD Korea Ltd, Seoul, Korea). After intubation, a radial artery catheter and an additional peripheral venous line were inserted. Anaesthesia was maintained with sevoflurane in 40% O_2_ and remifentanil infused at 0.05–0.15 µg kg^−1^ min^−1^. Controlled hypotension, with a systolic blood pressure < 100 mmHg, was used during maxillary manipulation. Red blood cells were transfused when the Hb fell below 8.5 g/dL.

### Data collection and outcome assessments

The primary outcome was the postoperative blood Hb level. Secondary outcomes included reticulocyte count, and reticulocyte production index, serum levels of iron, ferritin, transferrin, transferrin saturation, and total iron binding capacity (TIBC), and perioperative blood transfusion requirements. Reticulocyte production index was the reticulocyte percentage corrected for both the haematocrit and reticulocyte lifespan^20^. The reticulocyte count and reticulocyte percentage were measured automatically using the ADVIA 2120i analyser (Siemens Healthcare, Erlangen, Germany). All laboratory values were obtained before surgery and at 1 day, 2 weeks, and 4 weeks postoperatively.

### Statistical analysis

The sample size was calculated for the primary outcome (postoperative Hb). An Hb difference of > 0.8 mg/dL between iron and control groups was considered clinically relevant^11^. Twenty-six subjects were required in each group for a power of 80% at a significance level of 5%. To account for 10% dropout, we enrolled 29 in each group.

Demographic and intraoperative data are presented as number of patients, mean ± standard deviation, or median (interquartile range). Haematologic and iron parameters are expressed as estimated mean ± standard error values obtained from a linear mixed model. For continuous variables, the independent t-test was used to compare parametric data, and the Mann–Whitney U test was used for nonparametric data. The χ^2^ or Fisher’s exact test was used to evaluate categorical variables. Linear mixed models of fixed and random effects between groups were used to examine repeated measurements of haematologic and iron parameters. Intergroup comparisons of parameter changes over time included group-by-time interactions. Correlations between repeated measures were examined using an unstructured covariance matrix. Post hoc analysis was performed with Bonferroni correction to adjust for multiple comparisons. *P* values < 0.05 were considered statistically significant. Statistical analyses were conducted using SPSS 25.0 (IBM Corp., Armonk, NY, USA), R version 3.5.1 (R Foundation for Statistical Computing, Vienna, Austria), and SAS 9.4 (SAS Inc., Cary, NC, USA).

## Results

**Figure 1:**
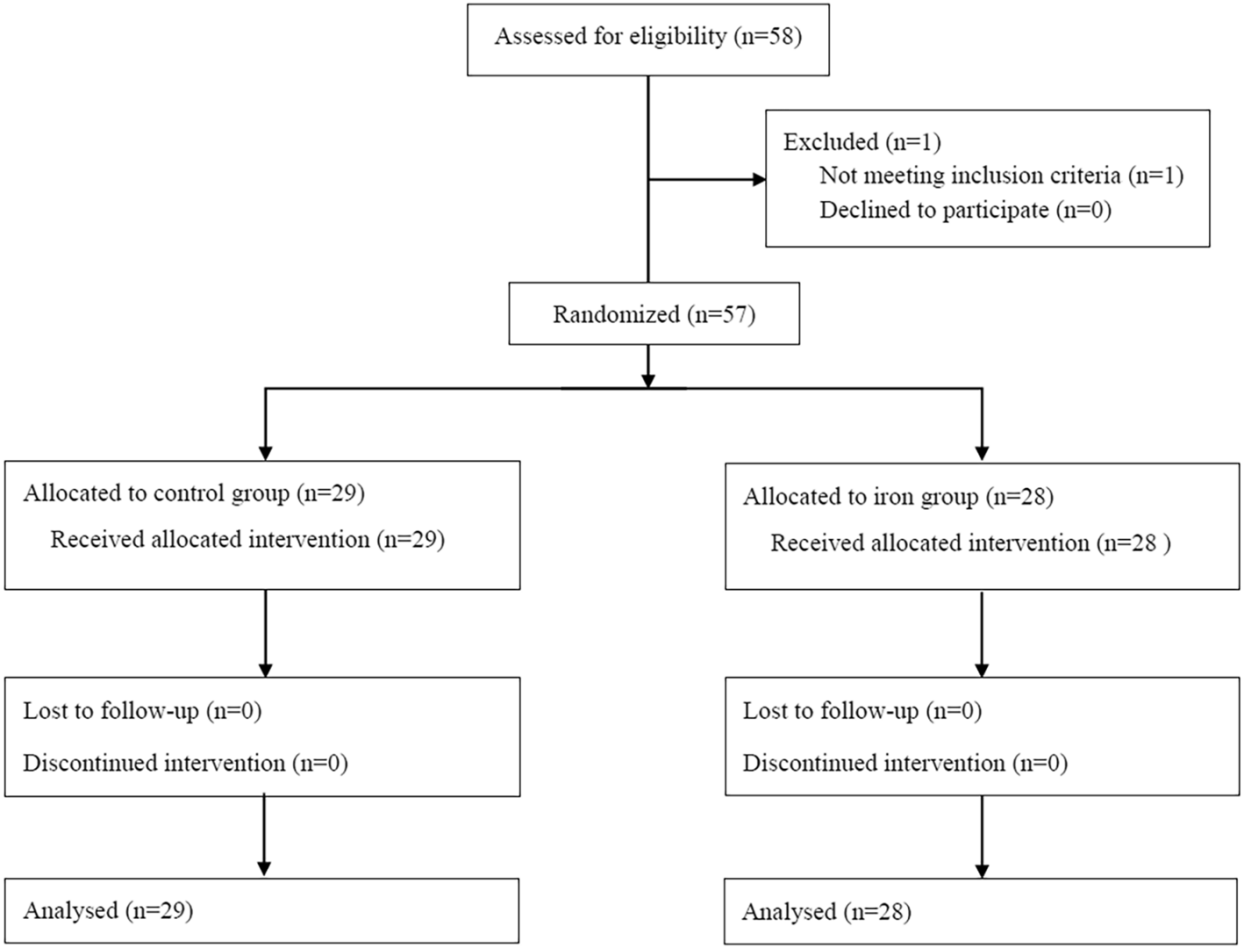
Flow diagram of the study.

Of the 58 subjects evaluated for eligibility, 57 were enrolled and assigned either to the iron (n = 28) or control group (n = 29) (Fig. 1). One subject was excluded due to allergic reaction to antibiotics in pre-anaesthesia room after informed consent. The remaining participants completed the study. As shown in Table 1, patient characteristics and intraoperative variables were not significantly different between groups. Before surgery, 26 patients (male:female, 4:22) had iron deficiency (serum ferritin < 30 µg/L) and 10 (male:female, 1:9) had a transferrin saturation of < 20% and serum ferritin of < 100 µg/L. Requirements of blood transfusion perioperatively did not differ significantly between groups: one (4%) individual in the iron group and three (10%) in the control group (*P* = 0.630).

**Table 1:** Patient characteristics and intraoperative data. Values are presented as median (interquartile range), mean $\pm$ standard deviation, or number of patients.

	Control group (n = 29)	Iron group (n = 28)	*P* value
Age (years)	23 (20–26)	22 (20–25)	0.681
Gender (male/female)	13/16	14/14	0.900
Height (cm)	168.5 $\pm$ 6.2	168.6 $\pm$ 7.3	0.957
Weight (kg)	61.0 (55.0–68.0)	59.5 (53.5–70.5)	0.661
Diagnosis			0.648
Apertognathia	0	1 (4)	
Facial asymmetry	15 (52)	12 (43)	
Prognathism	13 (45)	13 (46)	
Retrognathism	1 (3)	2 (7)	
Ferritin < 30 μg/L	12 (41)	14 (50)	0.514
Transferrin saturation < 20%	5 (17)	5 (18)	> 0.999
Operation time (min)	203 $\pm$ 37	211 $\pm$ 36	0.398
Anaesthesia time (min)	248 $\pm$ 38	251 $\pm$ 39	0.707
Intraoperative fluid intake (mL)	1935 $\pm$ 546	1863 $\pm 636$	0.645
Intraoperative bleeding (mL)	500 (300–650)	450 (325–700)	0.835
Patients required transfusion (n, %)	3 (10)	1 (4)	0.630

**Figure 2:**
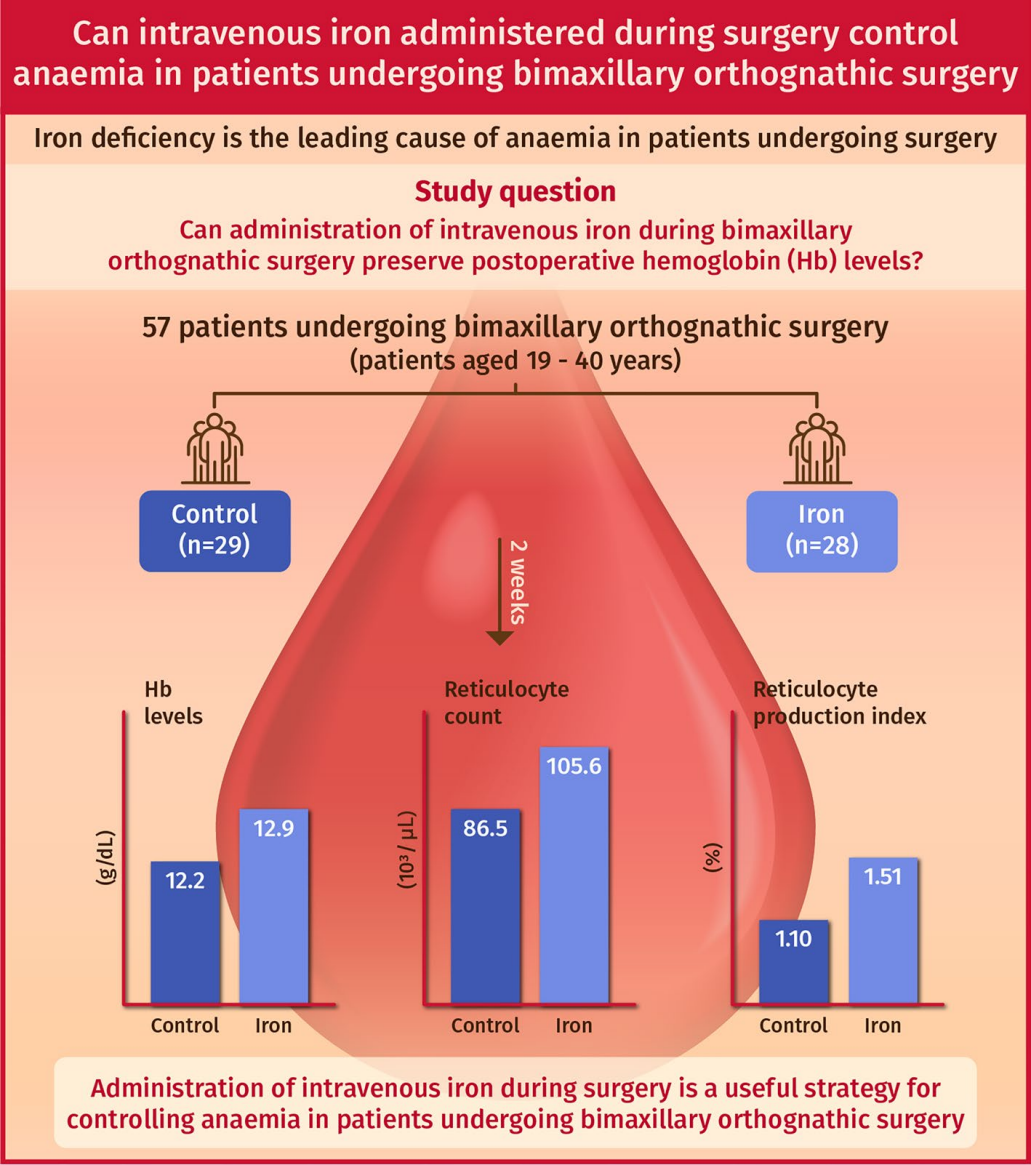
Infographic: Intraoperative iron administration accelerates the postoperative recovery of haemoglobin level.

At 2 weeks postoperatively, the Hb was higher in the iron group than in the control group but there was no statistical difference after adjustment for multiple testing (12.9 g/dL vs. 12.2 g/dL, *P* = 0.108) (Table 2). The reticulocyte count was higher in the iron group than in the control group at 4 weeks (95.1 × 10^3^/$\mu$L vs. 66.8 × 10^3^/$\mu$L, *P* = 0.045) after surgery. Corroborating these observations, the reticulocyte production index at 2 weeks postoperatively was higher in the iron group than in the control group (1.51 vs. 1.10, *P* = 0.018) (Fig. 2).

**Table 2:** Haematologic parameters.

	Control group (n = 29)	Iron group (n = 28)	Adjusted *P* value
**Haemoglobin base (g/dL)**	12.0 $\pm$ 0.3	12.3 $\pm$ 0.3	0.990
POD1	10.8 $\pm$ 0.3	10.8 $\pm$ 0.3	> 0.999
2 weeks	12.2 $\pm$ 0.3	12.9 $\pm$ 0.3	0.108
4 weeks	12.7 $\pm$ 0.3	13.4 $\pm$ 0.4	0.408
**Reticulocyte count base (10**^**3**^**/**$\mu$**L)**	92.0 $\pm$ 4.8	86.3 $\pm$ 5.0	> 0.999
POD1	89.9 $\pm$ 4.7	86.0 $\pm$ 5.0	> 0.999
2 weeks	86.5 $\pm$ 4.9	105.6 $\pm$ 5.4	0.105
4 weeks	66.8 $\pm$ 6.8	95.1 $\pm$ 10.1	0.045
**Reticulocyte production index base (%)**	1.33 $\pm$ 0.09	1.15 $\pm$ 0.09	0.282
POD1	1.09 $\pm$ 0.09	1.01 $\pm$ 0.09	> 0.999
2 weeks	1.10 $\pm$ 0.09	1.51 $\pm$ 0.10	0.018
4 weeks	1.07 $\pm$ 0.14	1.55 $\pm$ 0.25	0.459

Values are presented as estimated mean $\pm$ standard error from linear mixed model. *POD* postoperative day. Adjusted P value indicates the Bonferroni-corrected P-value.

Both serum iron and transferrin saturation were higher in the iron group than in the control group at 1 day and 2 weeks postoperatively but were similar in the two groups at 4 weeks after surgery (Table 3). Serum ferritin was higher in the iron group than in the control group at 2 weeks and 4 weeks after surgery. As expected, TIBC and transferrin levels were higher in the control group than in the iron group at 2 weeks and 4 weeks postoperatively.

**Table 3:** Iron parameters.

	Control group (n = 29)	Iron group (n = 28)	Adjusted *P* value
**Iron base (**$\mu$**g/dL)**	104.0 $\pm$ 25.7	92.1 $\pm$ 25.7	> 0.885
POD1	31.8 $\pm$ 17.9	605.3 $\pm$ 25.7	< 0.001
2 weeks	55.9 $\pm$ 26.2	83.0 $\pm$ 28.4	< 0.001
4 weeks	73.0 $\pm$ 39.2	77.0 $\pm$ 22.1	> 0.999
**Ferritin base (μg/L)**	46.1 $\pm$ 19.8	57.9 $\pm$ 19.9	> 0.999
POD1	63.6 $\pm$ 29.8	101.1 $\pm$ 27.9	0.075
2 weeks	57.4 $\pm$ 23.0	495.2 $\pm$ 26.2	< 0.001
4 weeks	30.9 $\pm$ 27.6	276.7 $\pm$ 38.4	< 0.001
**Transferrin saturation base (%)**	39.0 $\pm$ 2.55	34.2 $\pm$ 2.60	> 0.777
POD1	14.9 $\pm$ 4.43	80.6 $\pm$ 4.02	< 0.001
2 weeks	18.4 $\pm$ 3.16	31.8 $\pm$ 3.71	< 0.001
4 weeks	19.7 $\pm$ 4.02	27.0 $\pm$ 5.92	0.459
**TIBC base (μg/dL)**	271.7 $\pm$ 12.6	273.3 $\pm$ 12.8	> 0.999
POD1	275.7 $\pm$ 22.2	838.6 $\pm$ 20.1	< 0.001
2 weeks	325.7 $\pm$ 15.7	264.0 $\pm$ 18.5	< 0.001
4 weeks	342.3 $\pm$ 20.1	266.5 $\pm$ 29.8	< 0.001
**Transferrin base (mg/dL)**	220.6 $\pm$ 6.25	222.4 $\pm$ 6.42	> 0.999
POD1	227.8 $\pm$ 9.10	213.3 $\pm$ 8.92	> 0.999
2 weeks	267.1 $\pm$ 7.17	223.0 $\pm$ 8.15	< 0.001
4 weeks	284.9 $\pm$ 8.47	219.2 $\pm$ 11.7	< 0.001

Values are presented as estimated mean $\pm$ standard error from linear mixed model. *TIBC* total iron binding capacity. Adjusted P value indicates the Bonferroni-corrected P-value.

The linear mixed model revealed that there were statistically significant between-group differences in changes in reticulocyte count, reticulocyte production index, iron, ferritin, transferrin saturation, TIBC, and transferrin during the 4 weeks following surgery (Table 4).

**Table 4:** Relative change in iron-related blood parameters in the 4 weeks following surgery in the iron group compared to the control group, assessed using a linear mixed model.

Parameter	*P*_Group×Time_
Haemoglobin (g/dL)	0.102
Reticulocyte count (10^3^/$\mu$L)	0.002
Reticulocyte production index (%)	0.002
Iron ($\mu$g/dL)	< 0.001
Ferritin (μ/L)	< 0.001
Transferrin saturation (%)	< 0.001
TIBC ($\mu$g/dL)	< 0.001
Transferrin (mg/dL)	< 0.001

*TIBC* total iron binding capacity.

No surgical complication or adverse reaction related to IV iron was observed. Postoperative hospital length of stay did not differ between the groups (3 days vs. 3 days, *P* = 0.326).

## Discussion

In this randomised clinical trial, intraoperative administration of IV iron maintained Hb in the postoperative period by increasing haematopoiesis and iron bioavailability.

Patient blood management (PBM) is a multidisciplinary concept that focuses on patient safety by minimising blood loss and optimising physiological tolerance of anaemia^21^. Iron is necessary for erythroid cell proliferation, in addition to Hb synthesis, as it functions as a cofactor for several enzymes, such as DNA replicases, DNA polymerases, and DNA helicases^22^. These enzymes are necessary for cell division of all cells, including haematopoietic stem cells necessary for erythropoiesis^23^. Because iron is an essential component of red blood cell production, management of iron reserves during the perioperative period is important. However, surgically induced inflammatory responses upregulate hepcidin synthesis, and hepcidin decreases iron bioavailability by inhibiting iron absorption from the gastrointestinal tract and preventing the release of stored iron^24^. These effects lead to hypoferremia and iron-restricted erythropoiesis, even when iron stores are normal^25^. IV iron provides a readily available source of iron^25^, creating a five-fold erythropoietic response to anaemia resulting from blood loss^26^. Besides preoperative anaemia management, there are many perioperative care and alternative PBM strategies, including antifibrinolytics (tranexamic acid), intraoperative autologous cell salvage, anaesthetic management (autologous normovolemic haemodilution, controlled hypotension, normothermia), reduced phlebotomy blood loss (smaller tubes, eliminate unnecessary testing), and point-of-care coagulation monitoring (thromboelastography)^27, 28^.

Low serum ferritin level before IV iron and transferrin saturation were the useful parameters of iron stores within the body. Serum ferritin < 30 µg/L alone, or < 100 µg/L in the presence of serum transferrin saturation < 20%, indicated iron deficiency^5^. Serum ferritin < 100 µg/L or transferrin saturation < 20% was recommended to detect the iron deficiency in patients with chronic heart failure, chronic kidney disease, and inflammatory bowel disease^29^. A preoperative ferritin < 100 μg/L may indicate insufficient iron stores to recover from a 3–4 g/dL decrease in Hb decrease^5^. Treating preoperative iron deficiency can decrease the need for perioperative blood transfusion and consequently improve outcomes^12, 30^. However, the presence of iron deficiency is often not evaluated before surgery in healthy patients. Unexpectedly, 91% of subjects in our study had inadequate iron stores for moderate to high blood loss surgery, as evidenced by a serum ferritin < 100 µg/L preoperatively. Females were especially affected: 73% had a serum ferritin < 30 µg/L, and 90% had a serum ferritin < 100 µg/L plus transferrin saturation < 20% preoperatively. Of note, these subjects were all healthy (ASA class 1), but they did not have adequate iron stores for moderate blood loss surgery.

In cases with inadequate iron stores or iron deficiency but not anaemia, iron supplementation before surgery with an anticipated blood loss of > 500 mL might be beneficial^4^. Preoperative assessment and treatment of iron deficiency may be particularly important in women. Because of their lower blood volume, women have higher transfusion rates than males, when undergoing the same type of surgery^31^. Females with a preoperative Hb of 12 g/dL are two times more likely to receive a blood transfusion than males with an Hb of 13 g/dL^5^.

A high reticulocyte count after surgery reflects an increased erythropoietic response to blood loss^11^. Reticulocyte production index is used to assess whether the bone marrow is responding appropriately to the presence of anaemia^20^. The reticulocyte count and reticulocyte production index were higher in the iron group than in the control group at 2 weeks and 4 weeks after surgery, indicating that the intraoperative IV iron increased haematopoietic activity.

During progressive iron depletion, laboratory results typically show reduced serum ferritin and iron levels and increased serum transferrin and TIBC values^20^. These findings were evident in the control group at 2 weeks postoperatively, as expected, but not in the iron group. IV iron has been previously shown to improve iron biochemical outcomes and haematopoietic response to severe anaemia and provide long-term normalisation of Hb levels^32^. Prior studies reported that IV iron administered 2 to 4 weeks before surgery decreased perioperative red blood cell transfusion rates and hospital length of stay^14^. Furthermore, IV iron administration less than 1 week preoperatively has been associated with improved outcomes^11, 16^. However, iron supplementation is not always administered before surgery, and unexpected bleeding may occur during the operation. In these situations, intraoperative iron supplementation appears to be a good alternative, as our results showed that, in the absence of iron supplementation before surgery, intraoperative IV iron promoted haematopoiesis and iron bioavailability.

This study has some limitations. We included only healthy subjects; thus, the effects of intraoperative IV iron in patients with chronic disease remain unknown. We excluded candidates with anaemia due to kidney disease because they had a decreased ability of erythropoietin production and the error between subjects could be large^33^. Healthy people have a good haematopoietic response to blood loss, which can underestimate the effect of intravenous iron. In patients whose iron availability is inhibited by chronic inflammation, intravenous iron facilitates the rapid replenishment of available iron and Hb levels, resulting in a greater haematopoietic effect than that in healthy individuals^29^. Although previous research suggested that supplemental iron improved markers of iron status and anaemia in patients with chronic disease and older persons^15, 34–36^, investigations of intraoperative iron use in this patient population should be done separately. Another potential limitation of our study was that the amount of bleeding during surgery was less than expected. However, because decreases in Hb are greater with larger volumes of blood loss, the postoperative difference in Hb levels between groups may have been even larger if blood loss was as high as anticipated. Lastly, we started administering intravenous iron immediately after induction to give it before the start of surgery. A certain amount of iron might be lost due to intraoperative bleeding. However, the results showed that iron parameters are not significantly affected by intraoperative blood loss. The preoperative anaemia or iron deficiency assessment has not been performed in some patients with a high risk of developing postoperative anaemia undergoing major surgery. In this case, even after anaesthesia, administration of iron before starting surgery would be effective for haematopoiesis based on our study.

In conclusion, intraoperative administration of IV iron maintains postoperative Hb values in patients undergoing bimaxillary orthognathic surgery by increasing iron bioavailability and haematopoiesis. Thus, intraoperative IV iron appears to be a useful strategy for blood management in patients undergoing operations with anticipated moderate to high blood loss.

## Data Availability

The data that support the findings of this study are available from the corresponding author on reasonable request.
